# Prevalence, measurement, and implications of frailty in stroke survivors: An analysis of three global aging cohorts

**DOI:** 10.1177/17474930231151847

**Published:** 2023-01-30

**Authors:** Peter Hanlon, Jennifer K Burton, Terence J Quinn, Frances S Mair, David McAllister, Jim Lewsey, Katie I Gallacher

**Affiliations:** 1 School of Health and Wellbeing, University of Glasgow, Glasgow, UK; 2 School of Cardiovascular and Metabolic Health, University of Glasgow, Glasgow, UK

**Keywords:** Frailty, stroke, epidemiology, mortality

## Abstract

**Background:**

Our understanding of the relationship between frailty and stroke, beyond the acute phase of stroke, is limited. We aimed to estimate the prevalence of frailty in stroke survivors using differing methods of assessment and describe relationships with stroke outcomes.

**Methods:**

We used data from three international population surveys (American Health and Retirement Survey/English Longitudinal Study of Ageing/Survey for Health and Retirement in Europe) of aging. Frailty status was assessed using the Fried frailty phenotype, a 40-item frailty index (FI) and the clinical frailty scale (CFS). We created estimates of frailty prevalence and assessed association of frailty with outcomes of mortality/hospital admission/recurrent stroke at 2 years follow-up using logistic regression models adjusted for age/sex. Additional analyses explored effects of adding cognitive measures to frailty assessments and of missing grip strength data.

**Findings:**

Across 9617 stroke survivors, using the frailty phenotype, 23.8% (n = 2094) identified as frail; with CFS, 30.1% (n = 2906) were moderately or severely frail; using FI, 22.7% (n = 2147) had moderate frailty and 31.9% (n = 3021) had severe frailty. Frailty was associated with increased risk of mortality/hospitalization/recurrent stroke using all three measures. Adding cognitive variables to the FI produced minimal difference in prevalence of frailty. People with physical frailty (phenotype or CFS) plus cognitive impairment had a greater risk of mortality than people with an equivalent level of frailty but no cognitive impairment. Excluding people unable to provide grip strength underestimated frailty prevalence.

**Interpretation:**

Frailty is common in stroke and associated with poor outcomes, regardless of measure used. Adding cognitive variables to frailty phenotype/CFS measures identified those at greater risk of poor outcomes. Physical and cognitive impairments in stroke survivors do not preclude frailty assessment.

## Introduction

Frailty is defined as an increased vulnerability to adverse outcomes following a stressor event such as illness.^1^ With an aging global population, frailty is increasingly important in healthcare policy and practice. Recent papers have described pre-stroke frailty but few have assessed frailty in stroke survivors.^2,3^ For a condition predominantly seen in older adults and associated with high burden of disability, this represents a fundamental research gap agreed by patients, carers, practitioners and researchers.^2,4^

An understanding of frailty prevalence in stroke survivors is essential for planning equitable stroke services. For example, the increasingly potent and complex medication regimes used for stroke secondary prevention and the drive for early discharge from hospital services may not be appropriate in advanced frailty. Severe frailty may alter the balance of risks and benefits, as has been shown in other disease areas.^5^

One reason for the limited data could be the perceived difficulty in assessing frailty in stroke survivors. Various methods for defining frailty are described with no consensus on the optimal approach. Commonly employed phenotypic measures of frailty consider physical characteristics such as grip strength or walking speed.^6^ Following a stroke, performance on these measures may be compromised due to the acute event rather than due to age-related progressive processes such as sarcopenia. It is unclear if these two routes to diminished strength have differing prognostic implications. Similarly, many of the frailty assessment tools major on physical aspects of health, but for stroke survivors, the neuropsychological effects of stroke are of equal or greater importance.^4,7^ Adding an assessment of cognition may improve the application of traditional frailty measures in the context of stroke.

## Aims

To estimate the prevalence of frailty in stroke survivors using three frailty measures: the frailty phenotype, the frailty index (FI), and the clinical frailty scale (CFS).
To assess the relationship between frailty and outcomes of mortality, hospitalization, and recurrent stroke.
To explore any impact of physical or cognitive impairment on the epidemiology or associations seen in stroke frailty.

## Methods

### Study population

We used data from three population surveys of aging: the American Health and Retirement Survey (HRS);^8^ the English Longitudinal Study of Ageing (ELSA);^9^ and the Survey for Health and Retirement in Europe (SHARE).^10^ These surveys share a common structure and have been harmonized to allow cross-country comparisons through the Gateway to Global Ageing project. Each survey consists of an interview and physical assessment in a subset of individuals.

We identified participants included in the physical assessment sample (undertaken in approximately half of waves 7–12 in HRS, waves 2, 4, and 6 in ELSA, and waves 1, 2, 4, 5, and 6 in SHARE) who reported having had a stroke. For each survey, we identified participants “baseline assessment” as the earliest wave that included physical assessment and self-report of stroke.

### Frailty assessment

We assessed frailty status at the earliest available wave in which each participant reported having had a previous stroke. We used three models of frailty: frailty phenotype,^6^ FI,^11^ and CFS.^12^

#### Frailty phenotype

The frailty phenotype is based on five criteria: low hand grip strength, slow walking pace, weight loss, low physical activity, and exhaustion. People with three or more criteria are considered “frail,” while people with one or two are classified “pre-frail.” The criteria used to assess the frailty phenotype were based on the original description by Fried adapted to the data available.^6^

Our cut-offs for low grip strength were based on the original Fried definition.^6^ In the SHARE survey, testing of grip strength was not undertaken if participants reported being unable to perform the measurement in one or both hands. For the main analysis, we imputed any participant who was unable to perform the grip strength measurement as having “low grip strength.” We performed two sensitivity analyses: first, all analyses were repeated imputing these same individuals as not having low grip strength; second, excluding participants who were unable to perform grip strength measurement. Participants who refused or who were missing for unknown reasons were treated as missing for all analyses and not imputed.

We assessed slow walking speed using self-report (to allow consistent assessment across all three data sources), defined as difficulty walking 100 m (or “one block” in the HRS). We assessed weight loss as a 5% reduction in weight from previous wave or a body mass index less than 18.5. We assessed low physical activity based on self-reported frequency of less than once weekly moderate or vigorous physical activity. We adopted the criteria used in previous operationalization of the frailty phenotype to assess exhaustion across the surveys.^13,14^ In HRS and ELSA, we assessed exhaustion using two questions: “I felt that everything was an effort” and “I could not get going.” A positive response to either question indicated exhaustion. In SHARE, we assessed exhaustion using the question “In the last month, have you had too little energy to do the things you wanted to do?”

#### FI

The FI,^11^ based on the accumulation of “deficits,” is a numerical count of deficits, each of which must meet specific criteria: deficits must be associated with poor health status, increase in prevalence with age, and be neither too rare (i.e. <1% prevalence) or ubiquitous among older people. An FI must contain at least 30 deficits; however, the deficits included can be adapted to the data available for a given sample. An individual’s FI is calculated as the total number of deficits present, divided by the total number of possible deficits.

We identified 40 deficits that were common to all three data sets (HRS, ELSA, and SHARE), according to the standard procedure described by Searle et al.,^15^ to include in the physical FI (Supplementary Materials). We described FI at pre-defined thresholds indicating robust (<0.12), mild (0.12–0.24), moderate (0.24–0.36), and severe frailty (>0.36).

#### CFS

The CFS^12^ quantifies the overall fitness or frailty of an older adult after evaluation by a clinician. The scale has nine grades, from very fit to terminally ill. To apply the CFS to our sample, we used survey questions similar to the original descriptors of levels 1 (very fit) to 8 (very severe frailty) of the CFS,^12^ as described previously.^16^ We collapsed the eight grades into robust (very fit to managing well), vulnerable or mild, moderate, and severe or very severe frailty to facilitate comparison with other measures.

### Cognitive variables

We identified measures of cognitive function common to all three surveys: orientation (participants asked to name day of the week, date of the month, month, and year) and delayed word recall (recall of 10 nouns after 5 min). In HRS, these questions were only asked to people ⩾65 years; therefore, we restricted the analyses of cognitive impairment to this age range for all surveys. In SHARE, participants were only asked cognitive questions at first interview. We therefore did not have data on cognitive function for those SHARE participants who had a first stroke after their initial interview (776/5089 participants aged ⩾65 years).

As cognition is not a feature of the frailty phenotype, we assessed its relationship by creating a six-category variable (robust, pre-frail or frail, with or without cognitive impairment). We did similar with the CFS, creating an eight-category variable. For these analyses, participants were identified as having cognitive impairment if they incorrectly identified two or more of day, date, month and year, and if they scored 6 or less (out of 10) on delayed recall. By contrast, the FI typically includes measures of cognitive function among the included deficits. We therefore added these five cognitive measures to the other 40 deficits already identified. We then analyzed the “physical” FI alongside the FI including cognitive measures.

### Outcomes

Outcomes were assessed for each participant at the wave after their “baseline” assessment. This was approximately two years following initial assessment.

We assessed all-cause mortality based on survey follow-up. Hospital admission was assessed by self-report. Recurrent stroke was based on the follow-up health assessment questionnaire. For ELSA, this was assessed as “how many strokes have you had since the last survey” (any number >0 indicating a positive response), and for HRS and SHARE, this was assessed as “have you had a further stroke since the last assessment” (answer “yes” indicating recurrent stroke). Transient ischemic attacks were not asked as part of any of the three survey assessments, and so did not form part of the outcome definition.

### Statistical analysis

We summarized baseline age, sex, and prevalence of frailty for all participants and for each survey separately. We used sample weights (provided by each survey) to account for probability of inclusion in the sample. This allows for calculation of more representative estimates of prevalence. We present both raw counts and percentages and percentages calculated using sample weights as well as household- and survey-level clustering (using the *survey* package in R).

We assessed outcomes using logistic regression models adjusted for age/sex. We described odds ratios and 95% confidence intervals both before and after the application of sample weights and adjustment for clustering within surveys. We restricted models for recurrent stroke and hospitalization to participants who survived until the first follow-up wave and therefore had follow-up data.

For participants aged ⩾65 years, we conducted all analyses using frailty measures including physical variables only, and then repeated the analyses with cognitive variables included. We performed sensitivity analyses to explore the impact of different methods of analyzing missing data for participants deemed unable to complete grip strength assessment.

## Results

**Figure 1.**
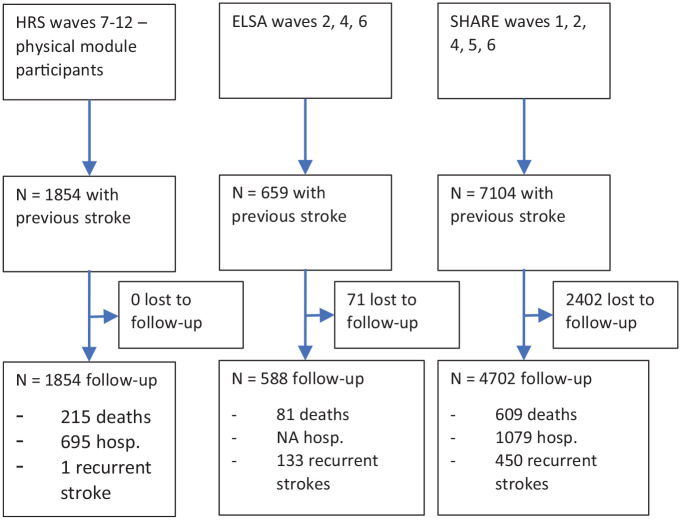
Flow diagram of data sources and participant follow-up.

We included 9617 people (1854/42,053 from HRS, 659/18,489 from ELSA, and 7104/120,047 from SHARE) who had reported a stroke (Figure 1).

**Figure 2.**
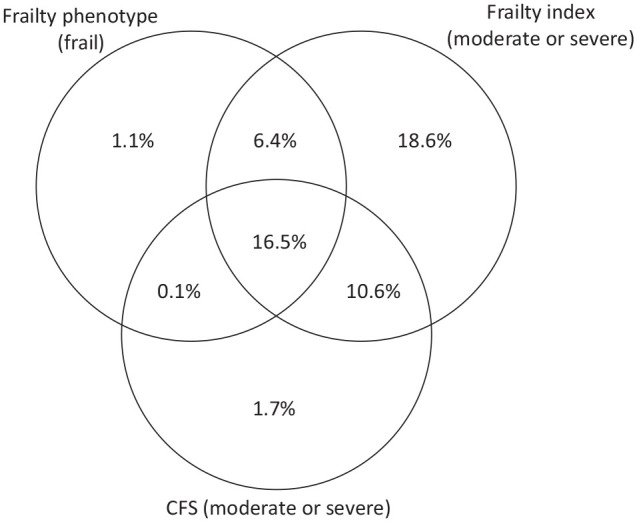
Venn diagram of participants identified as frail by each of the three scales. For the frailty index and CFS, all participants with “moderate” or “severe” frailty were included (FI > 0.24, CFS > 4).

Using the frailty phenotype definition (n = 8774 with complete data), 23.8% (n = 2094) of stroke survivors were identified as frail. Using the FI (n = 9446 with complete data), 22.7% (n = 2147) had moderate frailty and 31.9% (n = 3021) had severe frailty. Using the CFS (n = 9324 with complete data), 10.7% (n = 1000) had moderate frailty and 20.4% (n = 1906) had severe or very severe frailty (Supplementary Table). These proportions were similar after application of sample weights (Supplementary Materials). Figure 2 shows agreement between the measures, that is, the percentage of participants identified as frail by one, two, or all three measures.

In sensitivity analyses, after imputing people unable to complete grip strength assessment (n = 1857) as having no deficit in grip strength, the prevalence of frailty using the phenotype was 22.4%. When these participants were excluded from the analysis, the prevalence was 20.1% (Supplementary Materials).

Prevalence of cognitive impairment was low in the sample; however, using frailty phenotype, the prevalence was higher in the frail than robust group (3% of frail participants aged ⩾65 years, n = 153 as opposed to 0.2% of robust participants aged ⩾65 years, n = 10). Similar was found using the CFS (2.81% of frail participants ⩾65 years having cognitive impairment, n = 147 and 0.67% of robust participants ⩾65 years, n = 35). When cognitive variables were added to the FI, there was relatively little difference in the prevalence of frailty in people aged ⩾65 years (34.2% severely frail using physical variables only, 32.8% adding cognitive variables). At an individual level, there was some change in frailty categories in both directions after the addition of cognitive variables; however, FI values decreased more commonly than increased (Supplementary Materials).

**Figure 3.**
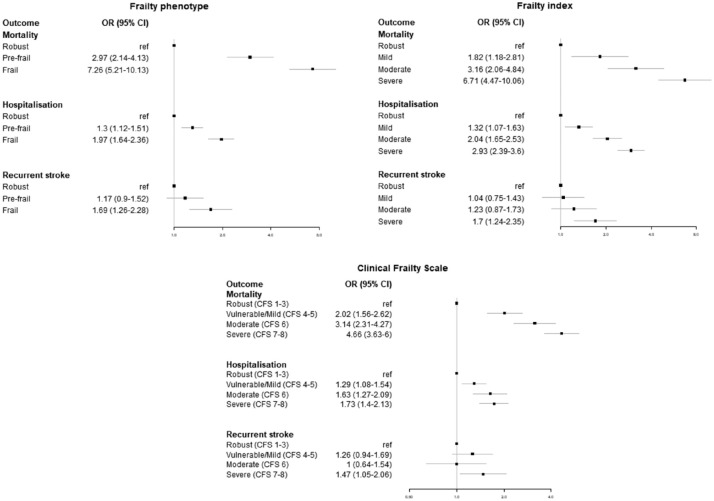
Relationship between frailty status and outcomes at 2 years follow-up, adjusted for age and sex.

Frailty was associated with increased risk of mortality, hospitalization, and recurrent stroke using the frailty phenotype, the FI, and the CFS (Figure 3). Results were similar in our sensitivity analyses regardless of method used for missing grip strength (Supplementary Materials). After applying sample weights, the effect size for recurrent stroke was greater.

**Figure 4.**
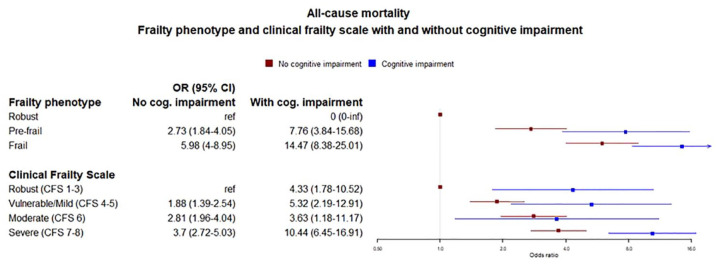
Relationship with outcomes—frailty phenotype with and without cognitive impairment.

People with frailty phenotype plus cognitive impairment had a greater risk of mortality than people with an equivalent level of frailty phenotype but no cognitive impairment (odds ratio (OR) = 5.84, 95% confidence interval (CI) = 3.92–8.70 for frailty without cognitive impairment, OR = 14.10, 95% CI = 8.19–24.29 for frailty with cognitive impairment). Similar was seen with the CFS (OR = 3.7, 95% CI = 2.72–5.03 for severe frailty without cognitive impairment, OR = 10.44, 95% CI = 6.45–16.91 for severe frailty with cognitive impairment) (Figure 4). For hospital admission and recurrent stroke, the smaller numbers of events in some categories led to wide confidence intervals with no clear relationship seen (Supplementary Materials).

**Figure 5.**
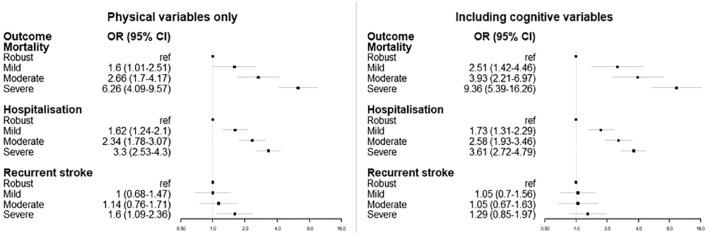
Relationship between frailty index and outcomes, before and after including cognitive deficits to the frailty index.

Comparing the relationship between the FI and all outcomes before and after adding the cognitive deficits, there were small differences in magnitude, but not direction, of effect (Figure 5).

## Discussion

In this large assessment of frailty, we found that frailty was common in stroke survivors, but prevalence varied by measure. Using the 40-item FI, 55% of stroke survivors were deemed frail or severely frail whereas only 24% were deemed frail when using the frailty phenotype and 31% were deemed moderately, severely, or very severely frail when using the CFS.

Frailty was associated with poor outcomes using all three measures. Those who were frail were more likely to be cognitively impaired than those who were robust. Adding cognitive variables into the assessments made little difference to frailty prevalence estimates. People with frailty plus cognitive impairment had a greater risk of mortality than people with an equivalent level of frailty but no cognitive impairment. Our sensitivity analyses on grip strength suggested that those unable to complete grip strength assessment were mostly frail by other criteria.

## Results in context

Several recent papers have described frailty in stroke, reporting high prevalence of frailty and associations between frailty and poor outcomes.^17^ These papers have tended to focus on acute stroke and so offer a measure of pre-stroke frailty. Our data show that patterns of high prevalence and poor outcomes remain apparent in a more chronic stroke survivor population. Method of frailty measurement in previous studies has been varied, one study formally compared agreement between measures, finding a fifth of their participants categorized as frail using both FI and frailty phenotype.^18^

Results around associations between frailty and post-stroke cognitive function have been mixed.^19,20^ Our findings that cognitive impairment was associated with an increased mortality risk may reflect cognitive impairment as a marker of increased stroke burden. There is some evidence in the context of dementia that frailty increases the clinical expression of dementia for an equivalent neuropathological burden.^21^ We lacked data on stroke severity to test such hypotheses with these data. We could not find any previous studies that have examined the association between frailty and recurrent stroke or hospitalization.

That many individuals were deemed frail by the FI but not by frailty phenotype or the CFS could be explained by the latter two being more dependent on functional assessments. All three measures were associated with worse health-related outcomes, validating their use as a measure of frailty after stroke. Finally, findings suggest that being unable to complete handgrip assessment should not exclude an individual from frailty phenotype assessment, and that excluding these individuals from frailty research risks introducing bias. Imputing low hand grip for these individuals is a pragmatic solution. These findings echo findings from a study of cognitive testing after stroke that suggested that those who cannot complete cognitive screening are likely to be cognitively impaired.^22^

## Implications

Identification of frailty can contribute to the provision of person-centered healthcare. Identification of frailty may shift the focus of stroke management away from intensive therapies toward social support and interventions to improve quality of life. However, there is current uncertainty over if and how the risks and benefits of specific treatments vary by frailty. This highlights the need to measure frailty more widely in clinical and research settings. Our findings suggest that the frailty measures presented are suitable for use in stroke survivor populations. Our findings allow researchers and clinicians to be more informed in their choice of measure; however, this may be a pragmatic decision based on feasibility. Increased knowledge about frailty measurement in stroke brings with it the potential for standardization of measurement, for example, the inclusion of frailty measurement in national stroke audits, and allows new avenues of stroke research, for example, retrospective derivation of frailty in previous clinical trials of stroke treatments.^23^

## Strengths and limitations

The large number of participants and wide range of demographic and frailty-related variables included in this study are key strengths. Application of sample weights suggested our sample was representative of the target population (older people who have had a stroke living in Europe and the United States). The number of participants with cognitive deficits was small, and this resulted in less precise estimates when examining health-related outcomes in that group. As with all observational studies, there were missing data, particularly data on height and weight. Data imputation was not conducted. We adapted the frailty phenotype and CFS to the data available and chose frailty variables that were available in all three data sets for the FI.

## Conclusion

Frailty is common after stroke, and measurement can provide important information for care planning. The FI, frailty phenotype, and CFS are valid measures of frailty after a stroke. The physical and cognitive impairments seen in stroke do not preclude frailty assessment, although people with frailty plus cognitive impairment are at greater risk of mortality than people with an equivalent level of frailty but no cognitive impairment.

## Supplemental Material

sj-docx-1-wso-10.1177_17474930231151847 – Supplemental material for Prevalence, measurement, and implications of frailty in stroke survivors: An analysis of three global aging cohorts Supplemental material, sj-docx-1-wso-10.1177_17474930231151847 for Prevalence, measurement, and implications of frailty in stroke survivors: An analysis of three global aging cohorts by Peter Hanlon, Jennifer K Burton, Terence J Quinn, Frances S Mair, David McAllister, Jim Lewsey and Katie I Gallacher in International Journal of Stroke
