# Supplementary material for: Prevalence, measurement, and implications of frailty in stroke survivors: An analysis of three global aging cohorts
Source: Int J Stroke. 2023 Jan 30;18(6):720–7. doi: 10.1177/17474930231151847 (PMC10311928; doi:10.1177/17474930231151847)

**Supplementary Material: Prevalence, measurement and implications of frailty in stroke survivors: an analysis of three global aging cohorts**

Table of Contents

[Data acknowledgement 2](#_Toc120864407)

[Deficits included in the frailty index 3](#_Toc120864408)

[Baseline frailty status – full sample 5](#_Toc120864409)

[Baseline frailty status adding cognitive variables – over 65 years only 5](#_Toc120864410)

[Descriptive baseline characteristics: before and after sample weights 7](#_Toc120864411)

[Comparison of frailty prevalence with different approaches to missing grip strength data 9](#_Toc120864412)

[Relationship between frailty and clinical outcomes: Comparison of approaches to missing grip strength data 10](#_Toc120864413)

[Relationship between frailty and clinical outcomes: Weighted versus unweighted results 11](#_Toc120864414)

[Frailty index categories with and without cognitive deficits 13](#_Toc120864415)

[Physical frailty with and without cognitive impairment: relationship with outcomes 14](#_Toc120864416)

[Frailty index with and without addition of cognitive measures: relationship with outcomes 16](#_Toc120864417)

# Data acknowledgement

This paper uses data from SHARE, HRS and ELSA. The SHARE data collection has been funded by the European Commission, DG RTD through FP5, FP6, FP7 and Horizon 2020 and by DG Employment, Social Affairs & Inclusion through VS 2015/0195, VS 2016/0135, VS 2018/0285, VS 2019/0332, and VS 2020/0313. Additional funding from the German Ministry of Education and Research, the Max Planck Society for the Advancement of Science, the U.S. National Institute on Aging and from various national funding sources is gratefully acknowledged (see www.share-project.org).

This paper also uses data from the HRS public use dataset. Produced and distributed by the University of Michigan with funding from the National Institute on Aging.

ELSA was developed by a team of researchers based at University College London, NatCen Social Research, the Institute for Fiscal Studies, the University of Manchester and the University of East Anglia. The data were collected by NatCen Social Research. The funding is currently provided by the National Institute on Aging in the US, and a consortium of UK government departments coordinated by the National Institute for Health Research. Funding has also been received by the Economic and Social Research Council.

# Deficits included in the frailty index

| Difficulty bathing | Yes = 1 , No = 0 |
| --- | --- |
| Difficulty dressing | Yes = 1 , No = 0 |
| Difficulty standing from chair | Yes = 1 , No = 0 |
| Difficulty walking across a room | Yes = 1 , No = 0 |
| Difficulty eating | Yes = 1 , No = 0 |
| Difficulty reaching/extending arms up | Yes = 1 , No = 0 |
| Difficulty using the toilet | Yes = 1 , No = 0 |
| Difficulty climbing stairs | Yes = 1 , No = 0 |
| Difficulty lifting/carrying (10 pounds) | Yes = 1 , No = 0 |
| Difficulty shopping for groceries | Yes = 1 , No = 0 |
| Difficulty preparing a hot meal | Yes = 1 , No = 0 |
| Difficulty taking medication | Yes = 1 , No = 0 |
| Difficulty managing money | Yes = 1 , No = 0 |
| Difficulty walking 100m | Yes = 1 , No = 0 |
| Difficulty getting in and out of bed | Yes = 1 , No = 0 |
| Difficulty using the telephone | Yes = 1 , No = 0 |
| Difficulty sitting for 2 hours | Yes = 1 , No = 0 |
| Difficulty stooping kneeling or crouching | Yes = 1 , No = 0 |
| Difficulty picking up a coin | Yes = 1 , No = 0 |
| Difficulty pushing or pulling a large object | Yes = 1 , No = 0 |
| Low physical activity | Yes = 1 , No = 0 |
| Self-rated health | Poor = 1  Fair = 0.75  Good = 0.5  Very good = 0.25  Excellent = 0 |
| Sad or depressed in last month | Yes = 1 , No = 0 |
| Enjoyment | Fails to mention any enjoyable activity = 1,  Mentions any enjoyment from activity = 0 |
| Difficulty sleeping | Yes = 1 , No = 0 |
| History of depression | Yes = 1 , No = 0 |
| Stroke | Yes = 1 , No = 0 |
| Hypertension | Yes = 1 , No = 0 |
| Heart disease | Yes = 1 , No = 0 |
| Cancer | Yes = 1 , No = 0 |
| Diabetes | Yes = 1 , No = 0 |
| Arthritis | Yes = 1 , No = 0 |
| Lung disease | Yes = 1 , No = 0 |
| High cholesterol | Yes = 1 , No = 0 |
| Fall in the last year | Yes = 1 , No = 0 |
| Dizziness | Yes = 1 , No = 0 |
| Shortness of breath | Yes = 1 , No = 0 |
| Hearing | Poor = 1,  Fair or good = 0.5,  Very good or excellent = 0 |
| Vision | Poor = 1,  Fair or good = 0.5,  Very good or excellent = 0 |
| Cataract | Yes = 1 , No = 0 |

# Baseline frailty status – full sample

|  | Combined (n=9617) | Separate cohorts | | |
| --- | --- | --- | --- | --- |
|  |  | HRS (n=1854) | SHARE (n=7104) | ELSA (n=659) |
| Age: mean (sd) | 71.1 (10.4) | 70.9 (11.4) | 71 (10.2) | 72.5 (9.8) |
| Sex (%) | \|  \|  \|  \|  \| \| --- \| --- \| --- \| --- \| |  |  |  |
| Female | 4793 (49.8%) | 974 (52.5%) | 3492 (49.2%) | 327 (49.6%) |
| Frailty phenotype (%) |  |  |  |  |
| Robust | 2351 (26.8%) | 442 (26.9%) | 1763 (26.9%) | 146 (24.8%) |
| Pre-frail | 4331 (49.4%) | 855 (52.1%) | 3213 (49.1%) | 263 (44.7%) |
| Frail | 2092 (23.8%) | 345  (21%) | 1568 (24%) | 179 (30.4%) |
| Missing | 843 | 212 | 560 | 71 |
| Clinical frailty scale |  |  |  |  |
| Robust | 3114 (33.4%) | 727 (46.4%) | 2289 (32.2%) | 98 (14.9%) |
| Vulnerable or mild | 3304 (35.4%) | 361 (23%) | 2657 (37.4%) | 286 (43.4%) |
| Moderate | 1000 (10.7%) | 185 (11.8%) | 714 (10.1%) | 101 (15.3%) |
| Severe | 1906 (20.4%) | 294 (18.8%) | 1438 (20.3%) | 174 (26.4%) |
| Missing | 293 | 287 | 6 | 0 |
| Frailty index (%) |  |  |  |  |
| <0.12 | 1392 (14.7%) | 140 (7.6%) | 1151 (16.5%) | 101 (15.3%) |
| 0.12-0.24 | 2906 (30.7%) | 478  (26%) | 2236 (32.1%) | 192 (29.1%) |
| 0.24-0.36 | 2147 (22.7%) | 452 (24.5%) | 1539 (22.1%) | 156 (23.7%) |
| >0.36 | 3021 (31.9%) | 772 (41.9%) | 2039 (29.3%) | 210 (31.9%) |
| Missing | 151 | 12 | 139 | 0 |

# Baseline frailty status adding cognitive variables – over 65 years only

|  | Combined (n = 6869) | HRS  (n = 1272) | SHARE  (n = 5089) | ELSA  (n = 508) |
| --- | --- | --- | --- | --- |
| Frailty phenotype |  |  |  |  |
| Robust no cognitive impairment | 1241 (24.5%) | 300 (26.6%) | 840 (24%) | 101 (22.5%) |
| Robust and cognitive impairment | 10 (0.197%) | 3 (0.266%) | 7 (0.2%) | 0 (0%) |
| Pre-frail no cognitive impairment | 2377 (46.9%) | 536 (47.6%) | 1644 (47.1%) | 197 (43.9%) |
| Pre-frail with cognitive impairment | 75 (1.48%) | 33 (2.93%) | 38 (1.09%) | 4 (0.891%) |
| Frail no cognitive impairment | 1213 (23.9%) | 232 (20.6%) | 844 (24.2%) | 137 (30.5%) |
| Frail with cognitive impairment | 153 (3.02%) | 23 (2.04%) | 120 (3.44%) | 10 (2.23%) |
| Missing | 1800 | 145 | 1596 | 59 |
| Clinical frailty scale |  |  |  |  |
| Robust no impairment | 1638 (31.3%) | 493 (48.9%) | 1069 (28.7%) | 76 (15%) |
| Robust with impairment | 35 (0.669%) | 18 (1.79%) | 14 (0.376%) | 3 (0.591%) |
| Vulnerable to mild no impairment | 1839 (35.1%) | 211 (20.9%) | 1417 (38.1%) | 211 (41.5%) |
| Vulnerable to mild with impairment | 43 (0.821%) | 10 (0.992%) | 29 (0.78%) | 4 (0.787%) |
| Moderate no impairment | 571 (10.9%) | 111 (11%) | 382 (10.3%) | 78 (15.4%) |
| Moderate with impairment | 30 (0.573%) | 5 (0.496%) | 24 (0.645%) | 1 (0.197%) |
| Severe no impairment | 932 (17.8%) | 145 (14.4%) | 662 (17.8%) | 125 (24.6%) |
| Severe with impairment | 147 (2.81%) | 15 (1.49%) | 122 (3.28%) | 10 (1.97%) |
| Missing | 1634 | 264 | 1370 | 0 |
| Frailty index  (physical only) (%) |  |  |  |  |
| <0.12 | 834 (12.4%) | 83 (6.56%) | 679 (13.7%) | 72 (14.2%) |
| 0.12-0.24 | 2030 (30.2%) | 351 (27.7%) | 1531 (30.9%) | 148 (29.1%) |
| 0.24-0.36 | 1564 (23.2%) | 334 (26.4%) | 1099 (22.2%) | 131 (25.8%) |
| >0.36 | 2301 (34.2%) | 498 (39.3%) | 1646 (33.2%) | 157 (30.9%) |
| Missing | 140 | 6 | 134 | 0 |
| Frailty index (plus cognitive) (%) |  |  |  |  |
| <0.12 | 756 (12.7%) | 37 (2.92%) | 675 (16.2%) | 44 (8.66%) |
| 0.12-0.24 | 1967 (33%) | 378 (29.8%) | 1439 (34.4%) | 150 (29.5%) |
| 0.24-0.36 | 1279 (21.5%) | 347 (27.3%) | 790 (18.9%) | 142 (28%) |
| >0.36 | 1954 (32.8%) | 507 (40%) | 1275 (30.5%) | 172 (33.9%) |
| Missing | 913 | 3 | 910 | 0 |

# Descriptive baseline characteristics: before and after sample weights

|  |  | Overall |  | HRS |  | SHARE |  | ELSA |  |
| --- | --- | --- | --- | --- | --- | --- | --- | --- | --- |
|  |  | Raw | Weighted | Raw | Weighted | Raw | Weighted | Raw | Weighted |
| Total |  | 9617 |  | 1854 |  | 7104 |  | 659 |  |
| Age | Mean | 71.1 | 71.1 | 70.9 | 70.1 | 71 | 71.2 | 72.5 | 72.1 |
|  | SD | 10.4 | 10.7 | 11.4 | 11.0 | 10.2 | 10.7 | 9.8 | 10.5 |
| Sex | Male | 4824 (50.2%) | 50.3% | 880 (47.5%) | 50.6% | 3612 (50.8%) | 49.8% | 332 (50.4%) | 50.6% |
|  | Female | 4793 (49.8%) | 49.7% | 974 (52.5%) | 49.4% | 3492 (49.2%) | 50.2% | 327 (49.6%) | 49.4% |
| Frailty phenotype | Robust | 2298 (26.2%) | 26.4% | 442 (26.9%) | 28.5% | 1710 (26.1%) | 25.6% | 146 (24.8%) | 25.1% |
|  | Pre-frail | 4340 (49.5%) | 48.6% | 855 (52.1%) | 53.2% | 3222 (49.2%) | 47.7% | 263 (44.7%) | 45.1% |
|  | Frail | 2136 (24.3%) | 25% | 345 (21%) | 18.3% | 1612 (24.6%) | 26.8% | 179 (30.4%) | 29.8% |
|  | Missing | 843 |  | 212 |  | 560 |  | 71 |  |
| Physical frailty index | Robust | 1392 (14.7%) | 13.1% | 140 (7.6%) | 8.9% | 1151 (16.5%) | 15% | 101 (15.3%) | 15.5% |
|  | Mild | 2906 (30.7%) | 28% | 478 (26%) | 26.1% | 2236 (32.1%) | 30% | 192 (29.1%) | 27.9% |
|  | Moderate | 2147 (22.7%) | 23.8% | 452 (24.5%) | 24.5% | 1539 (22.1%) | 23.2% | 156 (23.7%) | 23.6% |
|  | Severe | 3021 (31.9%) | 35.1% | 772 (41.9%) | 40.5% | 2039 (29.3%) | 31.7% | 210 (31.9%) | 33% |
|  | Missing | 151 |  | 12 |  | 139 |  | 0 |  |
| Clinical frailty scale | Robust | 3114 (33.4%) | 29.5% | 727 (46.4%) | 47.2% | 2289 (32.2%) | 29.4% | 98 (14.9%) | 14.2% |
|  | Vulnerable or mild | 3304 (35.4%) | 34.7% | 361 (23%) | 23.2% | 2657 (37.4%) | 36.9% | 286 (43.4%) | 42.6% |
|  | Moderate | 1000 (10.7%) | 12% | 185 (11.8%) | 12% | 714 (10.1%) | 8.8% | 101 (15.3%) | 15.3% |
|  | Severe | 1906 (20.4%) | 23.8% | 294 (18.8%) | 17.6% | 1438 (20.3%) | 25% | 174 (26.4%) | 27.8% |
|  | Missing | 293 |  | 287 |  | 6 |  | 0 |  |

# Comparison of frailty prevalence with different approaches to missing grip strength data

| **Frailty phenotype level** | **Overall** | **HRS** | **SHARE** | **ELSA** |
| --- | --- | --- | --- | --- |
| **Main analysis: Grip strength imputed as ‘low’ if participant unable to complete** | | | | |
| Robust | 2351 (26.8%) | 442 (26.9%) | 1763 (26.9%) | 146 (24.8%) |
| Pre-frail | 4331 (49.4%) | 855 (52.1%) | 3213 (49.1%) | 263 (44.7%) |
| Frail | 2092 (23.8%) | 345 (21%) | 1568 (24%) | 179 (30.4%) |
| Missing | 843 | 212 | 560 | 71 |
| **Sensitivity analysis 1: Grip strength imputed as ‘normal’ if participant unable to complete** | | | | |
| Robust | 2378 (27.1%) | 442 (26.9%) | 1788 (27.3%) | 148 (25.2%) |
| Pre-frail | 4435 (50.5%) | 855 (52.1%) | 3314 (50.6%) | 266 (45.2%) |
| Frail | 1961 (22.4%) | 345 (21%) | 1442 (22%) | 174 (29.6%) |
| Missing | 843 | 212 | 560 | 71 |
| **Sensitivity analysis 2: Participant excluded if unable to complete grip strength (complete case analysis)** | | | | |
| Robust | 2307 (28.9%) | 442 (26.9%) | 1719 (29.7%) | 146 (25.7%) |
| Pre-frail | 4081 (51.1%) | 855 (52.1%) | 2967 (51.3%) | 259 (45.6%) |
| Frail | 1604 (20.1%) | 345 (21%) | 1096 (19%) | 163 (28.7%) |
| Missing | 2700 | 212 | 2397 | 91 |

# Relationship between frailty and clinical outcomes: Comparison of approaches to missing grip strength data


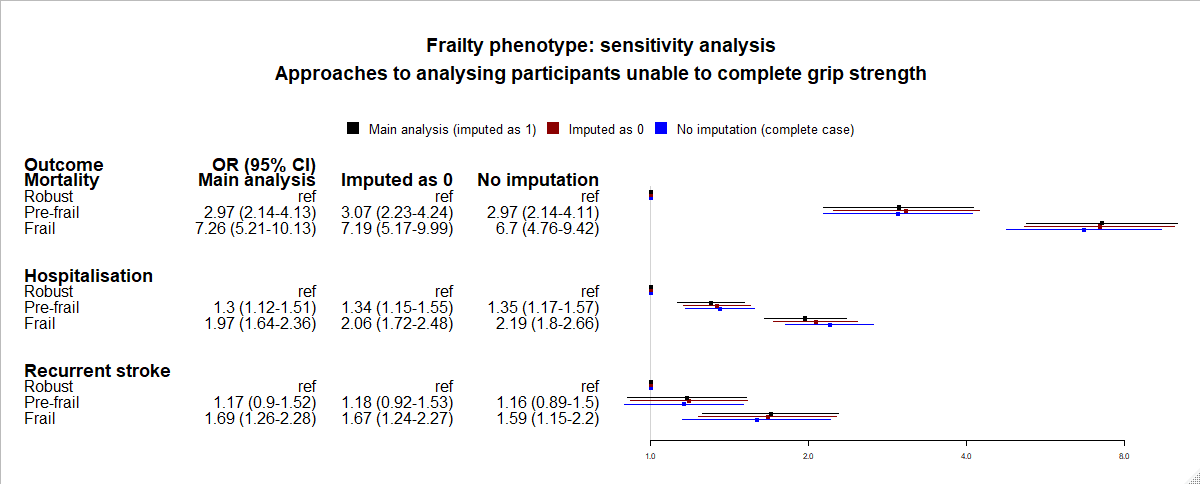


# Relationship between frailty and clinical outcomes: Weighted versus unweighted results


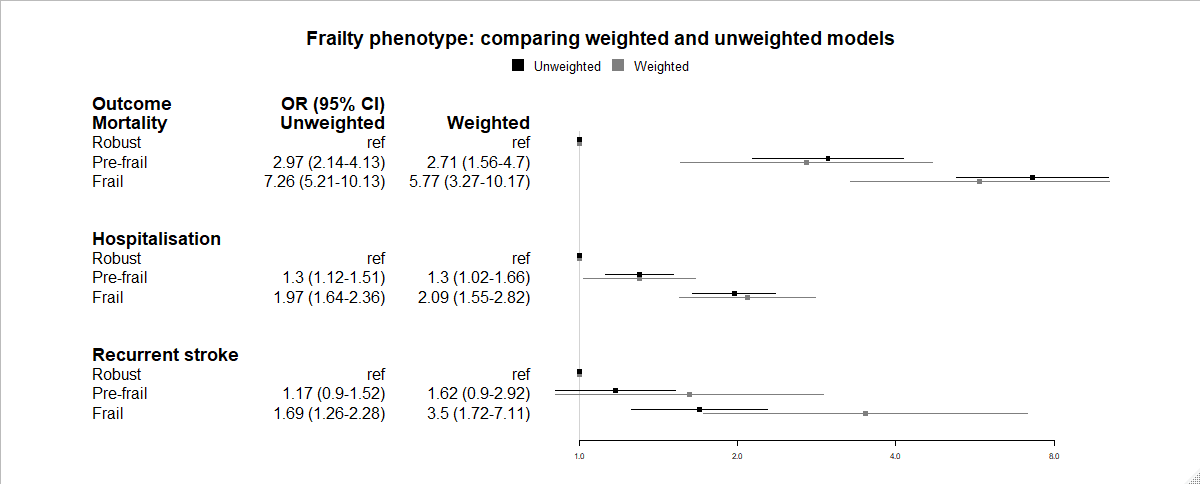


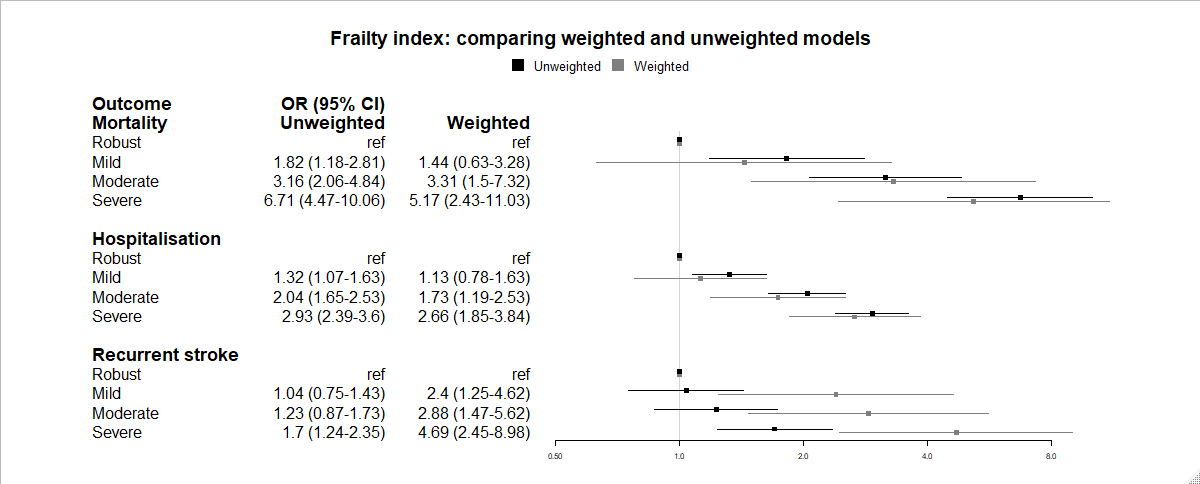


# Frailty index categories with and without cognitive deficits

|  | | Physical frailty index | | | |
| --- | --- | --- | --- | --- | --- |
|  |  | Robust | Mild | Moderate | Severe |
| Frailty index with cognitive deficits | Robust | 1079 | 247 | 0 | 0 |
|  | Mild | 172 | 2198 | 417 | 0 |
|  | Moderate | 0 | 115 | 1399 | 219 |
|  | Severe | 0 | 0 | 100 | 2445 |

# Physical frailty with and without cognitive impairment: relationship with outcomes


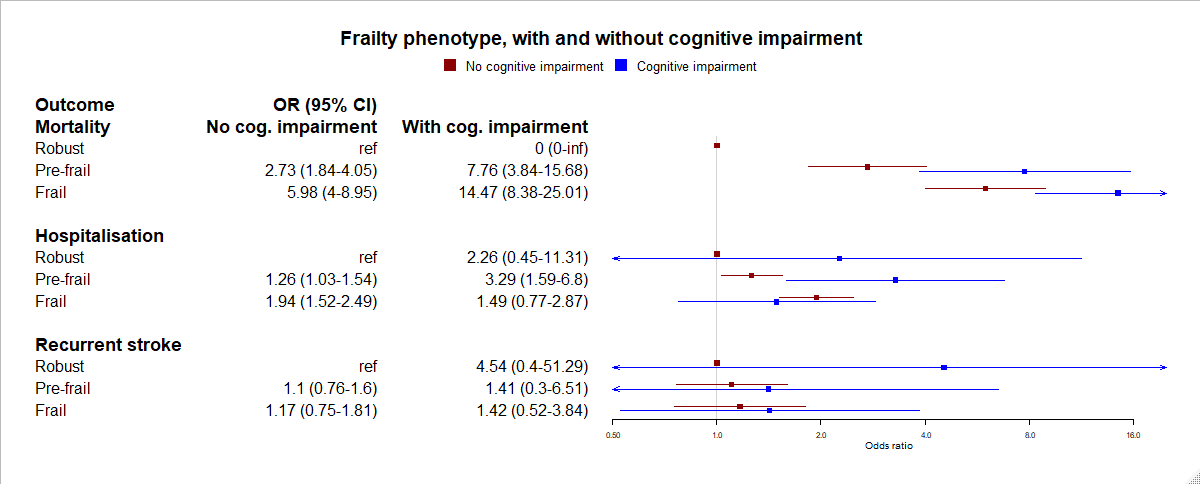


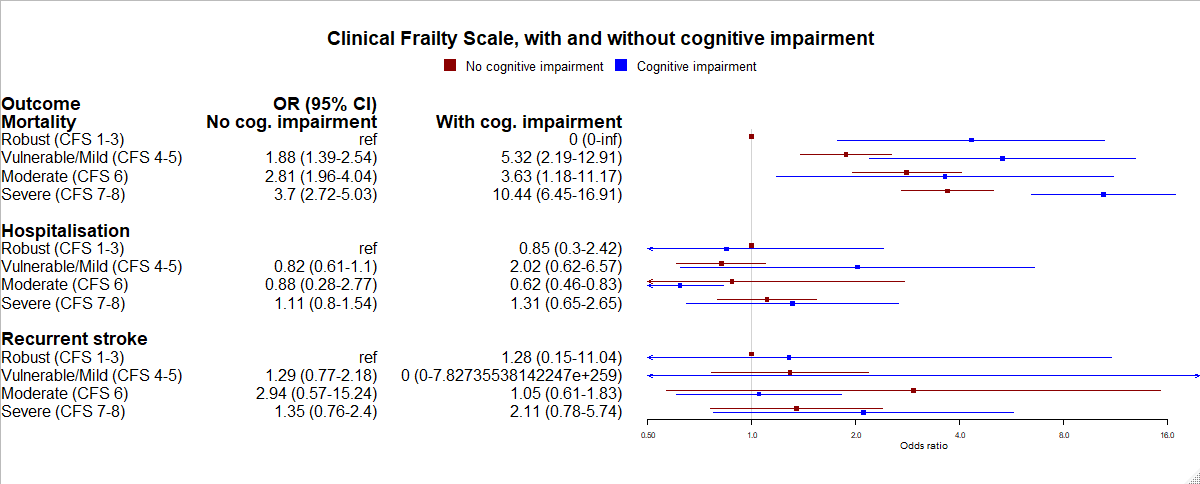


# Frailty index with and without addition of cognitive measures: relationship with outcomes


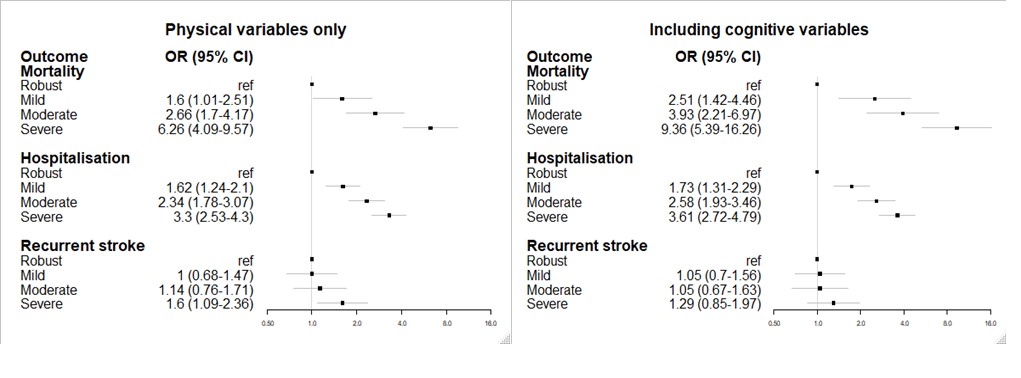

Supplement: sj-docx-1-wso-10.1177_17474930231151847 – Supplemental material for Prevalence, measurement, and implications of frailty in stroke survivors: An analysis of three global aging cohorts [file sj-docx-1-wso-10.1177_17474930231151847.docx]
